# A Case of Steroid-Responsive Severe Pneumonia Following a Recent COVID-19 Infection in a Patient With Pediatric Autoimmune Neuropsychiatric Disorders Associated With Streptococcal Infection

**DOI:** 10.7759/cureus.26785

**Published:** 2022-07-12

**Authors:** Seyedmohammad Pourshahid, Sara Khademolhosseini, Badri Giri, Moises Cossio, Edmundo Rubio

**Affiliations:** 1 Pulmonary and Critical Care, Virginia Tech Carilion School of Medicine, Roanoke, USA; 2 Department of Internal Medicine, Icahn School of Medicine at Mount Sinai, Queens Hospital Center, New York, USA

**Keywords:** immune reconstruction inflammatory syndrome, organizing pneumonia, post-covid pneumonia, steroid responsive pneumonia, multi-system inflammatory syndrome

## Abstract

A twenty-two-year-old woman with a history of pediatric autoimmune neuropsychiatric disorders associated with streptococcal infection (PANDAS) on rituximab presented with fever, abdominal pain, and worsening shortness of breath requiring supplemental oxygen via nasal cannula one month after a severe acute respiratory syndrome coronavirus 2 (SARS-CoV2) infection from which she was minimally symptomatic and had recovered. Radiographic studies revealed bilateral patchy consolidations interspersed with ground-glass opacities (GGO). She was started on antibiotics for presumed community-acquired pneumonia with no improvement. Echocardiography revealed preserved biventricular function and a suspected intracardiac mass. A cardiac magnetic resonance imaging (CMRI) revealed myocarditis and no intracardiac mass. Fever persisted and oxygen requirements increased from FiO2 0.4 to 1.0. Repeat CXR showed subtotal left hemithorax opacification. Bronchoscopic samples showed a negative Gram stain and an unremarkable cell count differential. In view of this and given her lack of response to antibiotics with worsening respiratory status, high-dose steroids were started. She improved rapidly, and six days later she was off oxygen. Transbronchial biopsies showed benign parenchyma with some intra-alveolar fibrin deposition with no definitive evidence of viral cytopathic effect, vasculitis, or diffuse alveolar damage (DAD). Follow-up imaging in the pulmonary clinic revealed improvement of prior airspace disease with some new migratory opacities that completely resolved after 12 weeks. Pulmonary function tests and repeat CMRI were normal three months after discharge. Multisystem inflammatory syndrome in adults (MISA), post-covid organizing pneumonia (OP), and immune reconstitution inflammatory syndrome (IRIS) are rare and potentially steroid-responsive causes of pneumonia, which were in our differential diagnosis. It is imperative to consider the rare possibility of steroid-responsive pneumonia-like MISA, post-COVID-OP, and IRIS in patients with worsening respiratory symptoms following a recent SARS-CoV 2 infection.

## Introduction

Persistent post-coronavirus disease 2019 (COVID-19) syndrome is a heterogeneous pathologic entity causing persistent physical and cognitive sequela. This may involve many systems, such as the pulmonary, cardiovascular, and immune systems, with various presentations [1].

Multisystem inflammatory syndrome in adults (MISA), post-COVID organizing pneumonia (OP), and immune reconstitution inflammatory syndrome (IRIS) are rare and steroid-responsive causes of pneumonia, which should also be considered in post-COVID-19 syndrome.

The Centers for Disease Control (CDC) defined MISA as patients 21 years or older who were hospitalized for more than a day or died of a febrile illness and who presented with a minimum of three clinical criteria, of which one must be primary. The primary clinical criteria included rash with non-purulent conjunctivitis or severe cardiac disease like myocarditis, pericarditis, etc. New-onset neurological symptoms, shock, gastrointestinal symptoms, or a platelet count less than 150,000/μL were among the secondary clinical criteria. Laboratory criteria included a recent positive test for severe acute respiratory syndrome coronavirus 2 (SARS-CoV2) infection in addition to elevated levels of at least two inflammatory markers [2].

The majority of MISA cases reported in the literature were not identified by the primary clinical team. MISA is commonly underdiagnosed as most COVID-19-related admissions do not have a comprehensive clinical and laboratory assessment to screen for this syndrome [3].

Organizing pneumonia is a form of interstitial lung disease that is usually idiopathic. Infections, drugs, aspiration, malignancies, and rheumatological diseases are also known etiologies for the secondary OP. Regardless of etiology, OP usually presents with sub-acute shortness of breath and cough [4]. The radiological presentation is widely varied with patchy bilateral pulmonary densities ranging from ground-glass opacities (GGO) to consolidation. Subpleural or broncho-vascular distribution is usually present mainly in the middle and lower lung zones that could be migratory or evolve to a reverse halo sign [5].

Restoration of the immune system is associated with a wide array of diseases resulting from a dysregulated immune response against specific antigens. The restored immune response may cause immunopathology while eradicating an infection or producing an autoimmune response [6]. IRIS is an immune-mediated inflammation occurring usually while recovering from immunosuppression. While it is best described in patients with human immunodeficiency virus (HIV) infection, it can also occur in non-HIV patients with conditions associated with recovery from an immunocompromised condition. It is usually triggered by an immune response to an antigen or an exacerbation of an inflammatory disorder or both [7].

We present an interesting case that illustrates the need for careful evaluation of post-COVID-19 disease.

## Case presentation

A 22-year-old woman presented to the hospital with a week of fever, generalized weakness, malaise, abdominal pain, and worsening shortness of breath. She had a positive SARS-CoV2 polymerase chain reaction (PCR) test a month before admission with minimal symptoms. She did not seek medical care at the time. She also reported a loss of appetite, nausea, and a dry cough for one week.

She had a history of pediatric autoimmune neuropsychiatric disorders associated with streptococcal infections (PANDAS) and celiac disease with no prior surgeries. Pre-admission medications included rituximab, which was given every three months for PANDAs, cetirizine for seasonal allergies, and multivitamins. She was due for her next rituximab infusion by the time she presented for this admission. She is a college student without known environmental exposure and denied any smoking history or substance abuse.

**Figure 1:**
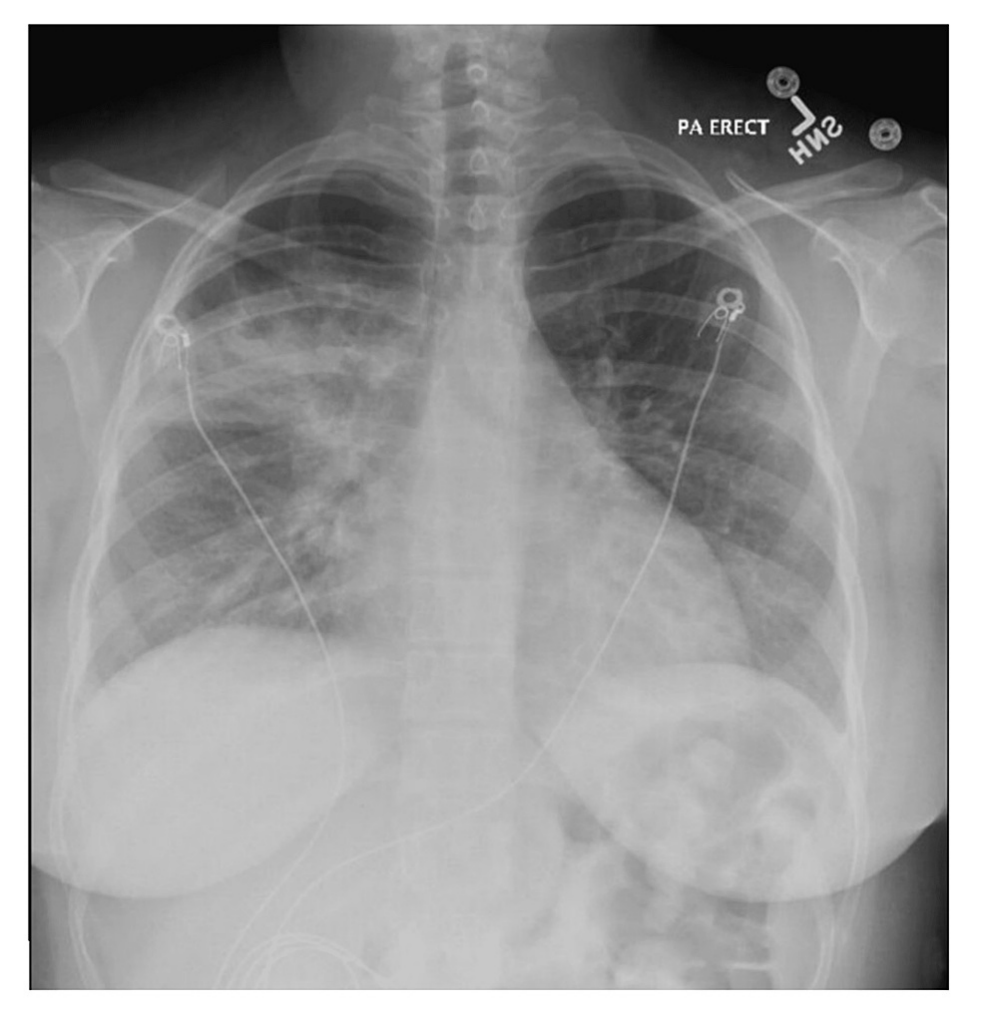
CXR on presentation showing multilobar pneumonia

In the emergency department, her temperature was 102 ˚F, her heart rate was 135 beats per minute, and her blood pressure was 104/59 mmHg with an oxygen saturation of 91% on room air. The physical exam showed right lower abdominal quadrant tenderness and diffuse bilateral crackles. A CXR showed multi-lobar consolidation changes without pleural effusion or pneumothorax (Figure 1).

**Figure 2:**
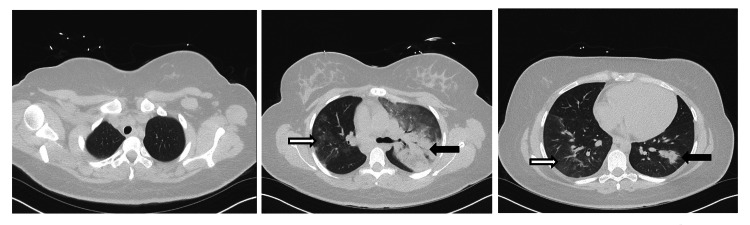
CT chest on presentation showing patchy, multilobar pulmonary consolidation and ground-glass opacities Left: upper lung zones, middle: mid lung zones at the level of left main bronchus, right: lower lung zones. Black arrows are pointing to the pulmonary consolidations, white arrows are pointing to the ground glass opacities.

Chest, abdomen, and pelvis computed tomography (CT) revealed significant consolidation with air bronchograms and GGO bilaterally (Figure 2). No intraabdominal pathology was noted, and the appendix was normal.

Remarkable laboratory results included C-reactive protein 8.26 mg/dL, D-dimer 0.76 FEU/mL, and a white blood cell count of 8.1 K/µL with no left-sided shift or increased band. SARS-CoV2 PCR and respiratory viral panel PCR were negative.

Empirically, ceftriaxone and azithromycin were started for suspected community-acquired pneumonia. Two days later, due to her immunocompromised status and considering her persistent fever, she underwent bronchoscopy with transbronchial biopsy and bronchoalveolar lavage (BAL) of the right upper lobe. The BAL had 4000 red blood cells/mm^3^, 1490 white blood cells/mm^3^ with 55% mononuclear cells, 43% lymphocytes, and 2% eosinophils. The BAL smear and culture were negative.

**Figure 3:**
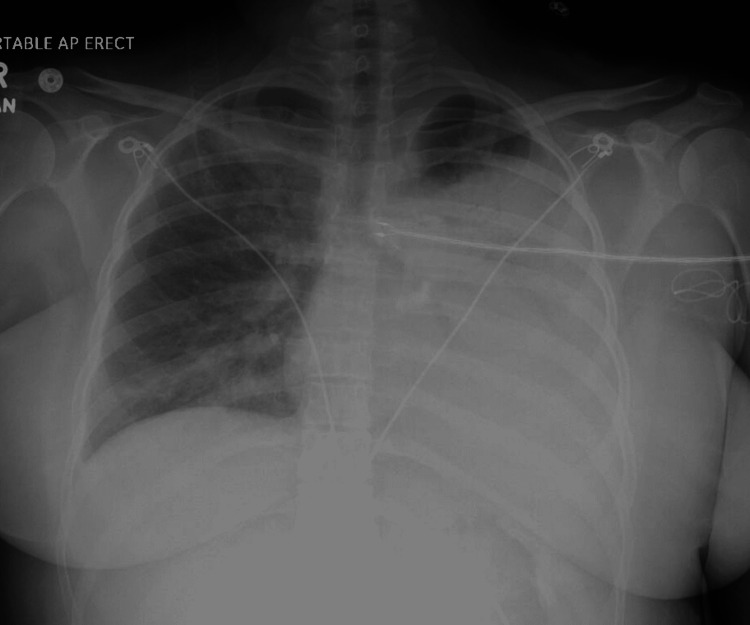
CXR after bronchoscopy showing near-complete opacification of the left hemithorax

After bronchoscopy, she continued to rapidly worsen with oxygen requirements increasing to a high flow nasal cannula at 60 liters per minute with FiO_2_ of 1.0 with saturations of 89% by the next day. The CXR revealed progression to diffuse alveolo-interstitial densities with signs of consolidation more pronounced in the left upper lobe and lateral aspect of the left lower lobe (Figure 3).

A CT angiogram showed extensive airspace opacification with air bronchograms mainly in the posterior aspects of the left upper and lower lobes, with no pulmonary embolism. An echocardiogram revealed preserved left ventricular function (65%) and an abnormal mobile density in the left atrium that was not attached to the mitral valve or septum (Video 1).

**Video 1:** Parasternal long view revealing an echogenic density in the left atrium LA: left atrium, LV: left ventricle

Differential diagnoses included artifact, interatrial septal aneurysm, or mass. Echo density was unchanged in a repeat echocardiogram with contrast, so cardiac magnetic resonance imaging (CMRI) was performed. This demonstrated mild biventricular cardiomyopathy, small pericardial effusion, small bilateral pleural effusion, and apical right ventricular hypokinesia with patchy areas of late gadolinium enhancement (LGE) consistent with myocarditis, with no intracavitary mass.

**Figure 4:**
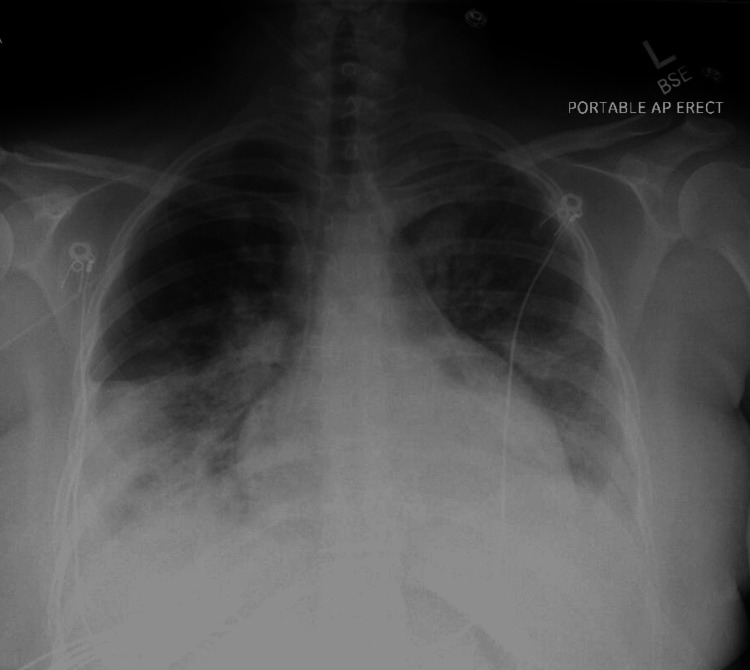
CXR before discharge showing significant improvement in aeration of the left lung but worsening consolidation of the right lung base

With no response to antibiotics and worsening pulmonary consolidation, she was started on methylprednisolone 125 mg IV every six hours. Within 24 hours, her oxygen requirement dropped to a FiO_2_ of 0.6, and by day 6, she was off oxygen. Transbronchial biopsies showed benign alveolar lung parenchyma with some intra-alveolar fibrin deposition with no definitive evidence of viral cytopathic effect, vasculitis, or diffuse alveolar damage (DAD). Small fragments of alveolated lung tissue with minimal chronic inflammation and foci of histiocytic proliferation were noted. The CXR before discharge showed significant improvement in aeration of the left lung and worsening pulmonary consolidations in the right lung base (Figure 4).

**Figure 5:**
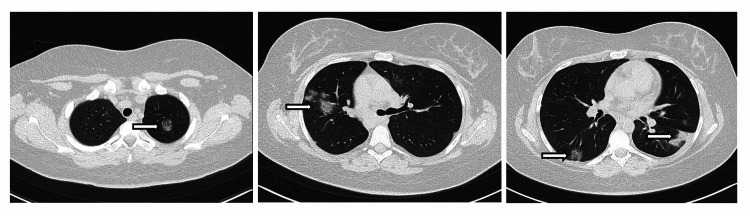
CT chest a month after discharge showing significant improvement in previously noted pulmonary consolidations. New bilateral patchy opacities were noted. Left: upper lung zones, middle: mid lung zones at the level of the left main bronchus, right: lower lung zones. White arrows are pointing to the new pulmonary opacities.

The patient was discharged home with a six-week taper of prednisone. Repeat chest imaging a month after discharge showed significant improvement in previously noted bilateral airspace disease. New patchy airspace opacities were noted (Figure 5).

**Figure 6:**
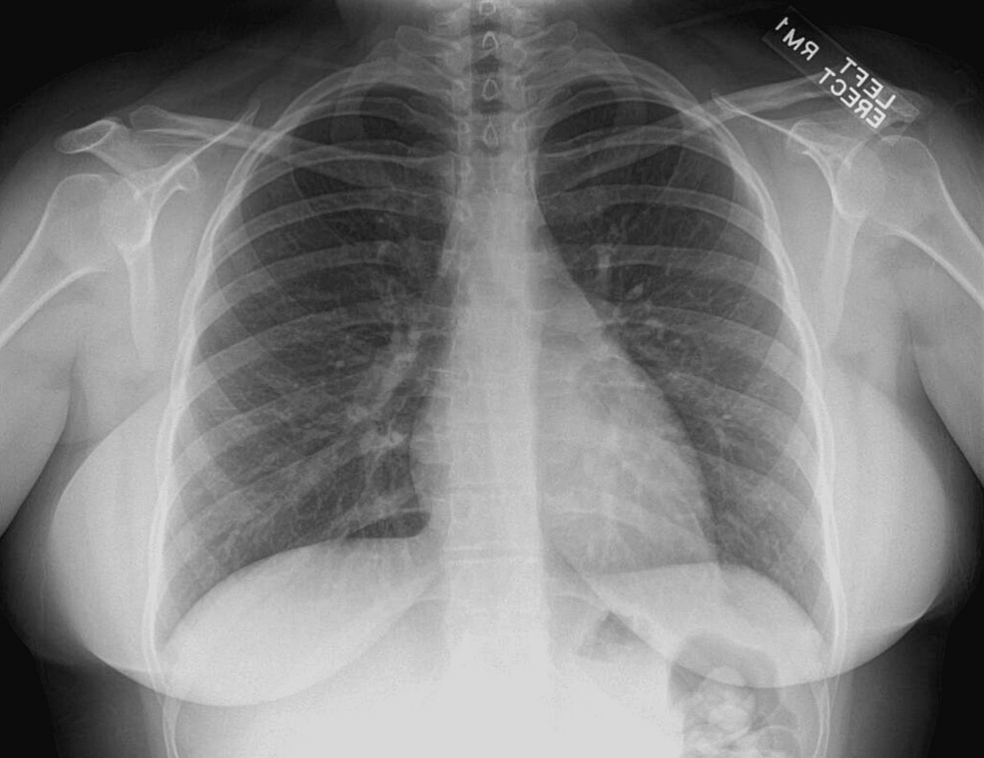
CXR twelve weeks after discharge showing resolution of pulmonary opacities after treatment with steroids

Finally, 12 weeks after discharge, chest imaging showed complete resolution of pulmonary opacities after treatment with steroids (Figure 6).

CMRI was repeated four months after discharge and revealed preserved LV and RV function. Patchy LGE spots in the RV were resolved. No intracardiac mass or thrombus was noted. Compared to the prior study, no evidence of myocardial inflammation was detected. The pulmonary function test was within normal limits with no evidence of impairment of gas exchange or obstructive or restrictive lung disease.

## Discussion

This patient developed rapidly progressive hypoxemic respiratory failure due to non-infectious pneumonia or pneumonitis. Incidentally, she also had evidence of myocarditis. She fulfills the clinical criteria for MISA considering her fever, evidence of myocarditis, and abdominal pain in her presentation. The laboratory criteria were also met based on prior SARS-CoV2 PCR and elevated inflammatory markers. Since she had neither conjunctivitis nor clinical evidence of severe cardiac illness, she would not have met the criteria for MISA had it not been for the incidental finding of an echocardiographic artifact that led to her CMRI. This is consistent with other reports of MISA in the literature that it rarely gets diagnosed by the primary team on clinical grounds. This is presumably due to a lack of comprehensive work-up to explore the presence of this syndrome in the majority of patients admitted for COVID-19 [3]. Despite all the difficulties in confirming this diagnosis, there is an increase in the number of reported cases of MISA [8]. It is important to consider MISA as a differential diagnosis in a wide variety of presentations in patients admitted during or shortly after a diagnosis of COVID-19.

She had a COVID-19 infection a month before admission, but her SARS-CoV2 PCR test was negative on this presentation. The initial recovery after her first positive test and the rapid progression of pulmonary opacities a month later made active COVID-19 an unlikely primary etiology for her symptoms. The negative respiratory viral panel and no growth in the cultures, in addition to the lack of response to empiric antibiotics, made an infectious process unlikely too.

The development of OP is in the differential diagnosis, but the progression seemed to be too rapid as OP usually has a subacute course. The transbronchial biopsy showed intra-alveolar fibrin deposition adjacent to normal lung structure with minimal chronic inflammation and foci of histiocytic proliferation that are consistent with post-COVID OP. However, this diagnosis usually requires excluding other possible causes like community-acquired pneumonia, hypersensitivity pneumonitis, chronic eosinophilic pneumonia, pulmonary malignancy, and DAD. We did not find any evidence that was consistent with eosinophilic pneumonia, hypersensitivity pneumonitis, malignancy, or DAD.

The natural history of post-COVID-19 recovery is not fully understood. Following SARS-CoV2 infection, a group of patients may continue to have ongoing symptoms of interstitial pneumonitis, sometimes in the form of OP [9]. Early treatment of post-COVID OP with steroids is associated with significant improvement of the symptoms [10]. However, this has become a point of controversy as the definition of non-resolving or slow-resolving pneumonia is arbitrary. Also, without a tissue confirmation for OP, there is an argument that expectant management would yield similar results, as they may require more time for recovery [11]. In this case, the patient had minimal symptoms during the time she was diagnosed with COVID-19 and she had a rapidly worsening course once she presented to the hospital one month later. This timeframe is not consistent with non-resolving post-COVID-19 pneumonia, while the migratory pulmonary opacities and slow worsening of constitutional symptoms are commonly seen in OP.

Since OP has a patchy distribution, identifying the predominant histopathologic patterns of OP like excessive granulation tissue in alveolar ducts and chronic inflammation of surrounding alveoli is needed as it could be just a bystander in many other pulmonary diseases [12]. In postmortem biopsy of patients dying of COVID-19 pneumonia, areas of diffuse alveolar damage transitioning to OP and acute fibrinous OP are frequently seen together [13].

Post-COVID OP is a complication that is vastly underrecognized. There are many reports of a strong association between COVID-19 and secondary OP requiring a high dose and prolonged course of corticosteroid therapy to achieve remission from respiratory failure. There is a lack of evidence that treating post-COVID OP with corticosteroids can prevent or reduce residual pulmonary fibrosis, especially after severe acute respiratory distress syndrome (ARDS) [4,14].

Another possibility to be considered in this case was that an exaggerated inflammatory response like IRIS could be triggered by the coronavirus infection for patients on immunomodulatory therapies. While IRIS is known as a phenomenon in HIV-infected patients after the initiation of antiretroviral therapy [15], there are reports of IRIS in non-HIV patients as well. Discontinuation or abrupt tapering of immunosuppressants, use of immune checkpoint antagonists in advanced stage malignancies, and withdrawal of anti-tumor necrosis factor-alpha antibodies are some of the potential triggers for non-HIV IRIS [16].

There are reports of immune reconstitution after SARS-CoV2 infection [17]. IRIS has various presentations, including interstitial pneumonia, type-one diabetes mellitus, thyroiditis, hepatitis, sarcoidosis, and various cutaneous manifestations [7]. The timing of IRIS presentations is widely variable, ranging from days to months depending on many factors, including infectious and noninfectious etiologies, the type of antigens, and pharmacologic agents. However, the majority of patients with IRIS become symptomatic within the first three months [18].

Additionally, there have been reports of multiple autoimmune conditions developing after COVID-19 infection, including autoimmune hemolytic anemia [19], autoimmune thrombocytopenia [20], Guillain-Barré syndrome [21], and IRIS [17]. An amplified monocyte activation from the effects of the SARS-CoV2 spike protein antibody response is one of the proposed mechanisms for post-COVID-19 IRIS. This response may lead to a proinflammatory cascade causing extrafollicular differentiation of B cells and activation of mucosa-associated T cells [22]. Despite much evidence suggesting the IRIS diagnosis, a transbronchial biopsy revealed mild chronic inflammation and organization.

For patients taking rituximab, the period of immunosuppression usually lasts six months to two years [23], but there are reports that B-cells have reappeared at 110 days, and it is unclear if a stimulus such as the one from a coronavirus infection like COVID-19 could trigger a more drastic and earlier response. Additionally, cases of early IRIS after rituximab therapy have also been reported, such as the case reported by Punch et al. [24].

Drug-induced pneumonitis was a possibility as well; however, she was only taking rituximab and cetirizine. Rituximab is a potential agent for the treatment of pneumonitis refractory to conventional treatments [25]. Although there are a few reported cases of rituximab-induced pneumonitis [26], the three-month delay after the last injection of rituximab and rapid progression in a few days are not consistent with drug-induced pneumonitis.

It is conceivable that her recent COVID-19 infection may have activated an exaggerated inflammatory response as the immunosuppression related to rituximab was subsiding. The rapidly progressing pulmonary consolidations and worsening respiratory symptoms under these circumstances should prompt the clinician to consider steroid-responsive-pneumonia like post-COVID OP, MISA, and IRIS-like reactions. Rapid institution of high-dose steroids seems to be the key to treatment. Whether her history of PANDAS may have further impacted her immune system, making her more prone to this exaggerated response is unclear.

## Conclusions

MISA and post-COVID OP are steroid-responsive and may be seen in some patients after SARS-CoV2 pneumonitis. IRIS is a rare differential diagnosis in rapidly progressing pneumonia that is not just ubiquitous in AIDS patients. Immunosuppressive medications may alter the course of post-COVID-19 organizing pneumonia and related inflammatory diseases affecting other systems.
